# Sarah Amy Hill

**Published:** 2007-02-27

**Authors:** Tim Hill

**Affiliations:** Publisher, Libertas Academica

**Figure:**
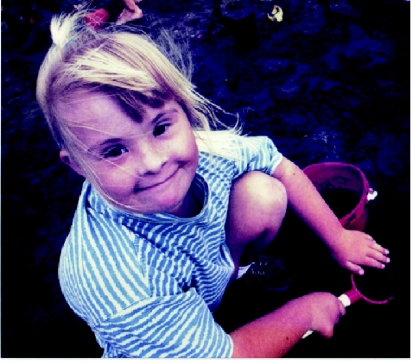


When Jim Lyons-Weiler and I discussed what we might do with an Open Access journal on *Cancer Informatics*, we both felt that it was important to avoid loosing focus on what the end-point of both the research and its dissemination is. In all its forms cancer is inevitably an unpleasant affliction that harms every aspect of the lives of sufferers and those who love them. Jim and I want this journal to focus on this fundamental reality. Therefore, we plan to dedicate every issue of the journal to a person and their experience with cancer.

I would like to dedicate this inaugural issue to my late daughter, Sarah Amy Hill. Sarah was born in 1983 in Auckland, New Zealand with Downs Syndrome. That, by itself, presented Sarah and those who loved her with many challenges. Sarah was somewhat slower than her contemporaries in reaching the developmental milestones, but armed with a strong will, charm, and a strong sense of humor she wore-down the obstacles in her path.

Starting school for Sarah at age 5 was an important moment and one that stimulated the mixture of excitement and nervousness familiar to all parents. Sarah loved school and was successful there, although she needed some extra help.

In that same year, Sarah began to show signs of loss of energy, and despite several trips to our physician the cause remained uncertain. It was only later when a rash appeared and blood tests confirmed that she was suffering from Acute Lymphoblastic Leukemia.

Sarah was aged just 6 years old, but she endured chemotherapy with strength beyond her years. On most days she was brave and matter-of-fact about it. On others she was a small frightened girl whose parents could do nothing to protect her from hair loss, chronic nausea and continuing general malaise.

As a family we were thrilled when Sarah went into remission. We had two wonderful years of good health, although our hearts were in our throats each time she attended the monthly clinic for blood tests.

On a black day in October 1994 we learned that Sarah had relapsed a second time, and that the outlook for remission was bleak. She slowly slipped away from us. As her health declined her will to live endured, and was an inspiration to all those who knew her.

Sarah died at 6pm on Sunday 22 January 1995 surrounded by those who loved her.

